# Synergistic Therapy of Celecoxib-Loaded Magnetism-Responsive Hydrogel for Tendon Tissue Injuries

**DOI:** 10.3389/fbioe.2020.592068

**Published:** 2020-11-27

**Authors:** Jingxin Wang, Likang Wang, Yueming Gao, Zhao Zhang, Xiaofeng Huang, Tong Han, Biyuan Liu, Yujie Zhang, Yilan Li, Lining Zhang

**Affiliations:** ^1^Department of Rehabilitation, Zhengzhou Central Hospital Affiliated to Zhengzhou University, Zhengzhou, China; ^2^Department of Rehabilitation Medicine, The Third Medical Centre, Chinese PLA General Hospital, Beijing, China; ^3^Department of Rehabilitation Medicine, The Second Medical Centre, Chinese PLA General Hospital, Beijing, China; ^4^Graduate School, Chinese PLA General Hospital, Beijing, China; ^5^Department of Endocrinology, The Second Medical Centre, Chinese PLA General Hospital, Beijing, China; ^6^Department of Epidemiology, School of Public Health Southern Medical University, Guangzhou, China; ^7^Department of Rehabilitation Medicine, The First Medical Centre, Chinese PLA General Hospital, Beijing, China

**Keywords:** tendon injury, celecoxib, macrophages, pulsed electromagnetic field, gait analysis

## Abstract

Tendon tissue injury is very common and always associated with pain, tissue swelling and even malformation if not treated on time. Traditional therapeutic strategies, such as cryotherapy, electrical therapy, ultrasound therapy and anti-inflammatory drug, are still unsatisfying. In this work, a synergistic therapy, based on the combination of celecoxib drug and pulsed electromagnetic field (PEMF) regimens, was developed for the treatment of tendon injury. This celecoxib-loaded magnetism-responsive hydrogel dressing (gelatin/Fe_3_O_4_/celecoxib) showed good biocompatibility and coordinated drug release behavior under the PEMF, which could effectively reduce the inflammatory reaction of macrophage cells with the incremental proportion of M2 macrophages at the injury site. CatWalk gait analysis further verified this synergistic effect of combination therapy for achieving the outstanding recovery of the injured tendon tissue. Thus, this magnetism-responsive hydrogel may represent a promising alternative strategy in clinics for promoting tendon healing.

## Introduction

Tendon injury is a common disorder that cause pain and motor dysfunction, which is one of the top 15 diseases with the highest incidence according to the statistics of the American Health System Diseases (Global Burden of Disease Study 2013 Collaborators, 2015). On account of the tendon pain, chronic degeneration or even fracture, it requires high cost of surgery and rehabilitation of 13,000–20,000 dollars per case with the long treatment period, thus seriously affecting the life qualities and activities of sufferers (Carr et al., 2015; Voleti et al., 2012). Even so, the newborn scar tissues after the tendon repair still display the abnormal structures, poor mechanical properties and high incidence of fracture after operation (Biressi et al., 2014), which brings about a huge economic burden to both families and society. Therefore, to thoroughly analyze the reasons in depth, effectively promote tendon healing and improve limb functions are great challenges for orthopedics and rehabilitation physicians.

Clinical studies show that inflammation exists during the repair of tendon injuries. A large number of inflammatory cells (macrophages and mast cells) were found in biopsies of human calcified tendinitis (Hackett et al., 2016). The biggest difference between adult tendon repair and embryonic tendon repair is the presence of an inflammatory response (Tang et al., 2014). Embryonic tendon repair is a regeneration mode without scar hyperplasia. Embryonic fibroblasts can reduce the recruitment of inflammatory cells to the injury site, and adult tendon repair belongs to scar healing. Tendon healing period includes the inflammatory, proliferation and remodeling processes. The main inflammatory cells in the process of tendon repair are macrophages with M1 type and M2 type (Voleti et al., 2012; Martinez and Gordon, 2014). M1 macrophages mainly exist in the early inflammatory time of tendon repair, which are beneficial for removing necrotic tissue and initiating tendon repair. However, it secretes pro-inflammatory cytokines of IL-1, TNF-α, and IL-6 that can induce the inflammation, scar formation, cells death and matrix degeneration to hinder the tendon repair. While M2 macrophages mainly exist in the late remodeling stage and produce the anti-inflammatory factor of IL-1 receptor antibodies (IL-1RA), IL-10, growth factors and extracellular matrix, which can effectively reduce inflammation and scars to promote the matrix remodeling and wound healing (Linderman et al., 2016).

Macrophage phenotype changes play an important role from the initial stage of inflammatory response to the fibrosis and remodeling stage of tendon injury (Sugg et al., 2014), and the macrophage infiltration and activation are initiating factors of tissue fibrosis. Previous studies have shown that the number of infiltrating macrophages is directly proportional to the degree of tissue fibrosis (Duffield, 2010). In the regulatory mechanism, the Janus kinase/signal transducer and activator of tran-ions (JAK/STAT) is the core pathway for inducing the macrophage activation and participating in the process of excessive fibrosis or remodeling after liver, kidney, cardiovascular and other tissue damages (Gombozhapova et al., 2017). Recently, the expression of STAT-6 was found in biopsy tissues of patients with advanced supraspinatus tendinitis, exhibiting the potential relation to the relief of tendon pain (Dakin et al., 2015), which indicated that the JAK/STAT-6 signaling pathway may be involved in macrophages tendon tissue and played an important role in the process of remodeling after injury, however, the role of JAK/STAT signaling pathway-mediated inflammation regulation in tendon remodeling was unclear so far as well as the regulation effects of cell polarization on the tendon remodeling after injury.

In the clinical work, many scholars try to use non-steroidal, anti-inflammatory and sugar cortical hormone drugs to inhibit the tendon disease inflammation. For example, celecoxib is the non-steroidal anti-inflammatory drug (COX-2 inhibitor) to inhibit plasma exudation causing inflammatory edema, which have shown that celecoxib inhibits the expression of inflammatory genes such as COX-2, NO, IL-6, MIP-1α, IL-1β through TLR2, JNK, and NF-κB, thus possessing an effective analgesic and anti-inflammatory activity in the treatment of tendon injury. However, two main disadvantages of conventional celecoxib are low concentration after oral administration and rapid drug clearance from the wound, which necessitates frequent large doses for maintaining effective drug concentration but with inevitable side effects. In addition, oral non-steroidal anti-inflammatory drugs are the prevailing chronic treatment option for tendon injury, however, their prolonged use is controversial due to their high risk to benefit ratio. Therefore, formulation of drugs in a controlled delivery system is of paramount importance to allow for prolonged drug residence time when long term treatment is required.

**SCHEME 1:**
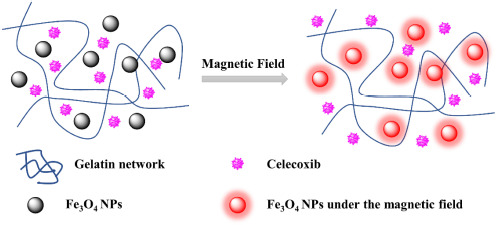
Schematic for formation of gelatin/Fe_3_O_4_/celecoxib system for the synergetic therapy of tendon tissue injuries.

Hydrogels are an attracting “soft-wet” material that consists plenty of water and distinctive 3D cross-linked polymeric network structures, which has aroused extensive attention due to its broad applications ranging from food chemistry and cosmetics to medical implants and scaffolds for tissue engineering (Liu H.Y.et al., 2020; Tang et al., 2020; Liu B.C.et al., 2020; Chen et al., 2019; Wang et al., 2020; Bao et al., 2020; Yang et al., 2021). Collagen, extracted from animals, is the main component of the extracellular matrix in mammalian tissues, such as skin, bone, cartilage, tendons and ligaments, which have been one of the most widely used natural hydrogel-like substances. Gelatin is a derivative of collagen and formed by breaking down the triple helix structure of collagen into single-stranded molecules, possessing no adverse reactions, no immunogenicity and good biodegradability *in vivo*. Therefore, gelatin hydrogels can be used as drug carriers to be applied or transplanted into an organism, thereby providing therapeutic benefits for achieving the controlled drug release system. Pulsed electromagnetic field therapy (PEMF) has already been a clinical method to accelerate tissue healing and recovery. Researches have suggested that PEMF could reduce pain via its effect on nitric oxide, calmodulin and/or opioid pathways, and it’s also a potential method to promote the drug absorption and tendon repair (Wanitphakdeedecha et al., 2017). In addition, pulsed electromagnetic field can change osmotic pressure and permeability, accelerate the blood circulation, reduce the colloid osmotic pressure of tissues, promote the exudate absorption, facilitate the protein transfer and relieve swelling and pain, which has a good therapeutic effect on the bloody and inflammatory swelling. In this work, we prepared a hydrated hydrogel dressing (gelatin/Fe_3_O_4_/celecoxib) with the synergistic therapies of celecoxib drugs and PEMF for the effective treatment of tendon tissue injury (Scheme 1). After tuning the pulsed electromagnetic field, the directional movement of embedded Fe_3_O_4_ nanoparticles can accelerate the celecoxib release, and meanwhile the generated heat can also loosen the gelatin network structures and further promote the drug release rate, exhibiting a kind of coordinated drug release behavior. In addition, in vivo implanting experiments were carried out to verify the biocompatibility and synergistic therapeutic effect. Combined with gait analysis, it was powerfully revealed that this synergistic therapy was significantly crucial and effectively used for tendon injuries repair and reshaping in clinical applications.

## Experimental Section

### Materials

Gelatin, ferric acetylacetonate (Fe(acac)_3_), 1,2-hexadecanediol and polyol medium triethylene glycol were purchased from J&K. Celecoxib was purchased from Dalian Meilun Biotechnology Co., LTD. All chemicals were used as received. All other chemicals were of analytical grade.

### Animals

Sprague Dawley rats (200 g) were purchased from the Animal Center of the Chinese PLA General Hospital. The animals were acclimatized for 1 week in an animal facility under controlled conditions of temperature (23 ± 3°C), relative humidity (55 ± 10%) and light (12/12 h light/dark, with no ultraviolet exposure). The animals had free access to a laboratory diet and ion-sterilized tap water. All experiments were performed in accordance with the guidelines of the care and use of laboratory animals of the Chinese PLA General Hospital, and experiments were approved by the animal ethics committee of the Chinese PLA General Hospital.

### Synthesis of Water-Soluble Fe_3_O_4_ NPs

Fe(acac)_3_ (1 mmol, 99%, Acros) and polyol medium triethylene glycol (30 mL) were mixed together and slowly heated to reflux (278°C) for 30 min under argon protection, producing a black homogeneous colloidal suspension. After cooled down to room temperature, 20 mL of ethyl acetate was added to the reaction solution, resulting in a black precipitation of magnetite nanoparticles which was then separated from the solution by a pulsed electromagnetic field. After being washed by ethyl acetate for three times, the precipitation was re-dispersed in water for further use.

### Preparation of the Gelatin/Fe_3_O_4_/Celecoxib Hydrogel

Firstly, gelatin was added into 10 mL of PBS solutions and heated to 40°C in an oil bath to form a transparent agarose solution, then celecoxib and Fe_3_O_4_ NPs were added into the solution. After cooling down to the room temperature, gelatin/Fe_3_O_4_/celecoxib hydrogel was formed in the suitable molds.

### Scanning Electron Microscopy (SEM)

The hydrogels were prepared as described above and then freeze-dried at −50°C for 48 h. Then the samples were carefully stuck onto the conducting resin with double-sided adhesive, and sputter-coated with a thin layer of Pt for 90 s to make the sample conductive before testing. SEM images were obtained at acceleration voltage of 5 kV on a JSM-6700F microscope (JEOL, Japan).

### *In vitro* Drug Release

Only one side of the hydrogel would touch the tissue during drug release process, so we employed the same release model as reported previously (Li et al., 2020). The hydrogel was prepared in a container with the diameter of 10 mm and height of 2 mm while the celecoxib was encapsulated inside the hydrogel. Then, the drug-contained hydrogel was immersed into the PBS (pH 7.4) and the solutions were collected at special intervals of time. During each interval, the magnetic field (pulsed magnetic therapy apparatus, 301-M9) was applied for 30 min. The collected solution at different time were tested using the HPLC system. The release without the PEMF was set as control.

### *In vitro* Cytotoxicity

*In vitro* cytotoxicity assays of gelatin/celecoxib and gelatin/Fe_3_O_4_ hydrogel were evaluated through the mouse embryos osteoblast precursor cells (MC3T3-E1) by the CCK-8 assay. Firstly, extraction of the hydrogel was obtained by immersing the hydrogel into the 1 mL of Dulbecco’s modified Eagle medium (DMEM). The MC3T3-E1 mouse fibroblasts were plated in 96-well cell culture at a density of 10^4^ cells/well, incubated with the extraction under 5% CO_2_ incubator at 37°C for 1 and 3 days and then changed to 100 μL of fresh DMEM and 10 μL of CCK-8. Subsequently, the cells were incubated at 37°C for another 4 h in a CO_2_ incubator. Then the cell viability was evaluated by comparing the absorbance of measured solutions using micro-plate reader at 450 nm. The final results were assumed to be the means of triplicate.

### Confocal Laser Scanning Microscopy (CLSM) Observation

MC3T3-E1 cells were seeded into 24-well culture plates at a density of 8,000 cells per well and incubated for 24 h at 37°C in humidified air containing 5% CO_2_. By using complete DMEM containing 10% FBS, 50 IU mL^–1^ penicillin and 50 IU mL^–1^ streptomycin, the culture media were replaced by extraction of the hydrogel. After cultivating for prescribed time intervals, MC3T3-E1 cells were washed by cold buffer solution for three times and immersed with 4% paraformaldehyde solution for 0.5 h at room temperature to fix the cell configuration. Then 4,6-diamidino-2-phenylindole (DAPI) was used for cellular staining and the process continued for 20 min. Finally, MC3T3-E1 cells were washed by buffer solution and directly transferred to glass culture dish for CLSM observation.

### Animal Model of Achilles Tendon Rupture

After the experimental animals were stabilized for 1 week, fifty SD rats were randomly divided into five groups of control (not treated), model (placebo treated), gelatin/celecoxib, gelatin/Fe_3_O_4_ and gelatin/Fe_3_O_4_/celecoxib hydrogel, which were anesthetized with an intravenous injection of pentobarbital sodium (50 mg/kg) and sterile skin preparation. Then a transverse incision was made at 0.5 cm of the insertion point in the Achilles tendon of rats. During the operation, the skin was cut and attention was paid to the protection of blood vessels to prevent vascular damage after the lumen, exposing the Achilles tendon tissue. Finally, a 1–2 mm of transverse defect was made to establish the rupture model of the Achilles tendon. After treatment of the Achilles tendon, the wound was washed with normal saline. The broken end of the Achilles tendon was not treated, and the skin was sutured with needle and thread followed by intramuscular injection of penicillin 1 × 10^5^ U d^–1^ for three continuous days to prevent infection. Then, the injured rats were treated by the hydrogel dressing with and without the PEMF (0.3 T) and celecoxib. After treatment for 3, 7, 14, and 28 days, the rats were euthanized and the tendon tissues of rats were collected for H&E staining staining for the observation and analysis of daily activities, gait and wound healing.

### Gait Analysis Using CatWalkTM

The CatWalkTM system was used to perform a detailed analysis of gait. In brief, the rats were placed in the system on a glass plate in a darkened room and allowed to walk freely. Light beams from a fluorescent lamp were sent through the glass plate, and the light beams were completely reflected internally. When a paw touched the glass plate, the light beams were reflected downward.

Thirty-five rats were randomly divided into five groups according to the random number table method: control, model, gelatin/Fe_3_O_4_, gelatin/celecoxib and gelatin/Fe_3_O_4_/celecoxib, and the rats were treated with samples at the rupture of Achilles tendon. In our experiment, the gait patterns of seven rats from each group were recorded with three times and analyzed automatically using the CatWalk system. Gait patterns analyzed included standing phase time, swing phase time, footprints area, mean intensity and swing speed. After observation for 1, 3, 7, 14, and 28 days, the gait parameters were measured and selected for analysis with a CatWalk gait analyzer.

### Statistical Analysis

All quantitative data were statistically analyzed via the *t*-test. *P*-value (<0.05) was considered statistically significant for all analyses.

## Results and Discussion

### Characterization of Water-Soluble Fe_3_O_4_ NPs

**FIGURE 1:**
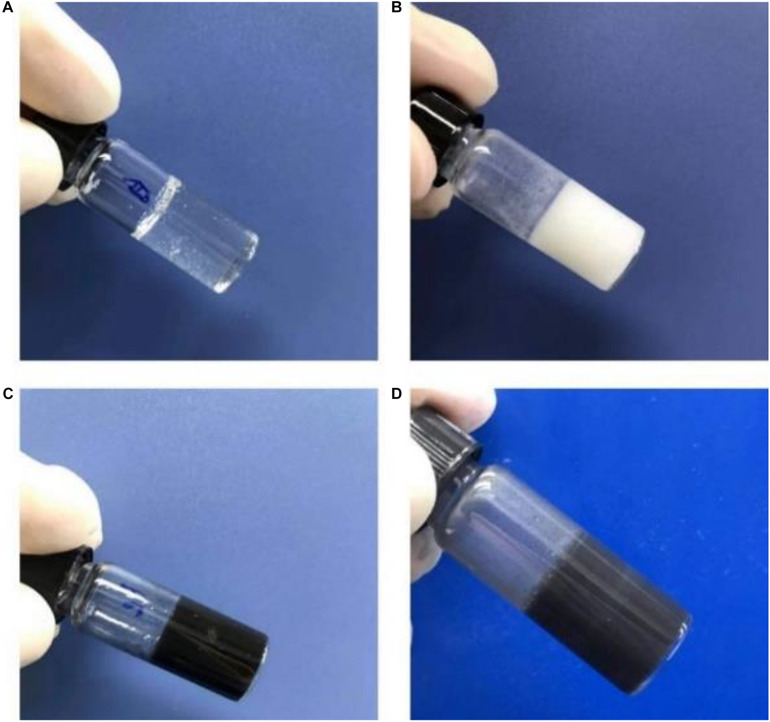
Photos of **(A)** gelatin, **(B)** gelatin/celecoxib hydrogel, **(C)** gelatin/Fe_3_O_4_ hydrogel and **(D)** gelatin/Fe_3_O_4_/celecoxib hydrogel.

Pulsed electromagnetic field therapy has become a clinical method to accelerate tissue healing and recovery. To combine magnet therapy with drug therapy, we firstly prepared water-soluble Fe_3_O_4_ nanoparticles (NPs) with the size of ca. 10 nm to provide an external magnetic field targeting function (Zhang et al., 2019). After the simple sol-gel transition process, we can easily obtain the transparent gelatin in Figure 1A. On account of the poor water solubility of celecoxib, gelatin/celecoxib hydrogel was white and opaque (Figure 1B) while the gelatin/Fe_3_O_4_ hydrogel was black and dense (Figure 1C). Therefore, black gray and opaque state in Figure 1D demonstrate the successful encapsulation of magnetic nanoparticles and drug molecules within the gelatin/Fe_3_O_4_/celecoxib hydrogel.

### Preparation of Drug-Loaded Magnetism-Responsive Composite Hydrogels

**FIGURE 2:**
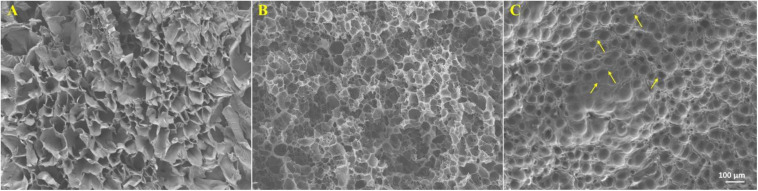
SEM images of **(A)** gelatin, **(B)** gelatin/celecoxib hydrogel and **(C)** gelatin/Fe_3_O_4_/celecoxib hydrogel. The arrows indicated the aggregated celecoxib and Fe_3_O_4_ NPs.

As shown in Figure 2, gelatin/Fe_3_O_4_/celecoxib hydrogels possessed porous structures with a dimension of ca. 60 μm, which was benefited for the nutrient exchange and drug delivery. Since the Fe_3_O_4_ NPs and celecoxib drugs were incorporated into the hydrogel *in situ*, we could observe some aggregates of the Fe_3_O_4_ and the celecoxib nanoparticles within the networks. In addition, the regular network structures indicated the favorable mechanics that will be feasible for the operative procedures.

### *In vitro* Drug Release

**FIGURE 3:**
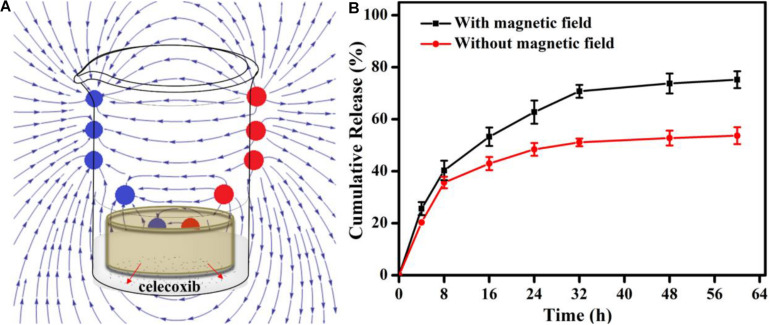
**(A)** Schematic showing the *in vitro* release experiment under the PEMF. **(B)** The release profiles of the dressing with (red) or without (black) the PEMF.

For synergistic effect of treatment, drugs were incorporated, of which the release can be controlled by extra magnetic field. Under the PEMF, the shaking of Fe_3_O_4_ NPs in the network could loosen the network to increase drug release rate, and the effect would be enhanced when the hydrogel was porous. In addition, heat can be generated to further accelerate the drug release (Xu et al., 2015; Liu Z.Y. et al., 2020). Considering that only one side of the hydrogel would contact the injured tendon tissue, gelatin/Fe_3_O_4_/celecoxib hydrogel was put in the receptacle with the diameter of 10 mm and height of 2 mm so that only one side was accessible to the release medium (Figure 3A). HPLC was used to record the release medium of celecoxib at different time. Figure 3B showed that the release speed under the magnet field was higher, and the total release amount under the pulsed electromagnetic field was 75.2 ± 3.5% compared to 53.7 ± 3.2% without magnetic field. Under the effect of pulsed electromagnetic field, the directional movement of embedded Fe_3_O_4_ nanoparticles can accelerate the celecoxib release, and meanwhile the generated heat can also loosen the gelatin network structures and further promote the drug release rate, presenting a coordinated drug release behavior and providing the possibility to combine the magnetic therapy and drug therapy for tendon repair.

### *In vitro* Biocompatibility

**FIGURE 4:**
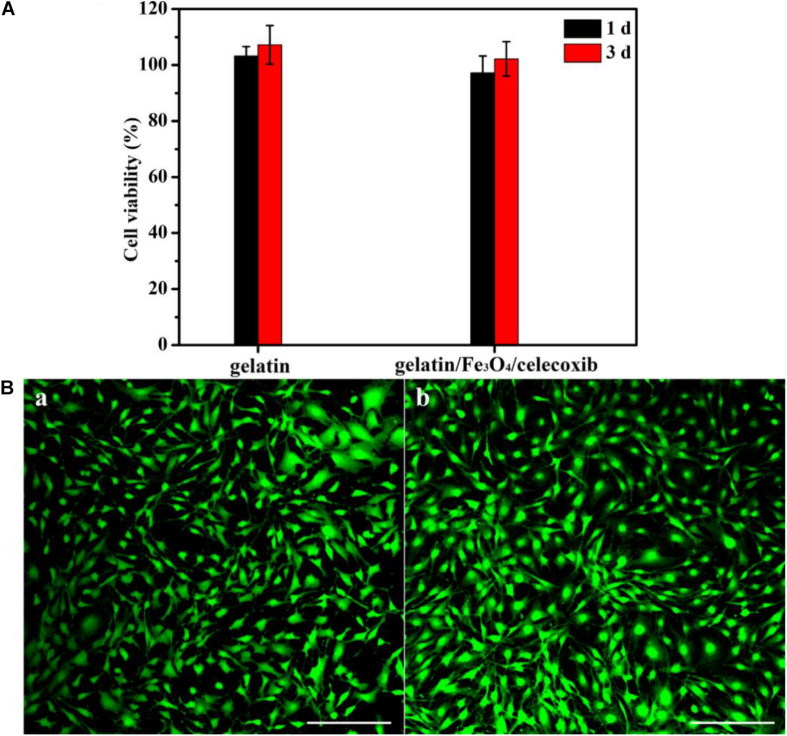
**(A)** Cell viability of gelation and gelatin/Fe_3_O_4_/celecoxib hydrogels to MC3T3-E1 cells. **(B)** CLSM images of **(a)** gelatin and **(b)** gelatin/Fe_3_O_4_/celecoxib hydrogel in MC3T3-E1 cells following 24 h incubation.

The *in vitro* cytotoxicity of gelation and gelatin/Fe_3_O_4_/celecoxib hydrogels was evaluated by CCK-8 assay. After incubation for 1-day, Figure 4A showed that the cell viability showed over 95% of cell viability even after 3 days of culture, demonstrating good biocompatibility. Then, Cell viability was intuitively observed using a live/dead staining measurement. The extraction of gelatin/Fe_3_O_4_/celecoxib hydrogels in DMEM medium were added directly to the MC3T3-E1 cells and DAPI dyes. After incubation for 24 h, they emitted the green fluorescents with healthy morphology (Figure 4B), revealing the excellent *in vitro* biocompatibility. Therefore, these gelatin/Fe_3_O_4_/celecoxib hydrogels could be utilized as potential candidates in various bio-applications.

### Animal Model of Tendon Tissue Injury

**FIGURE 5:**
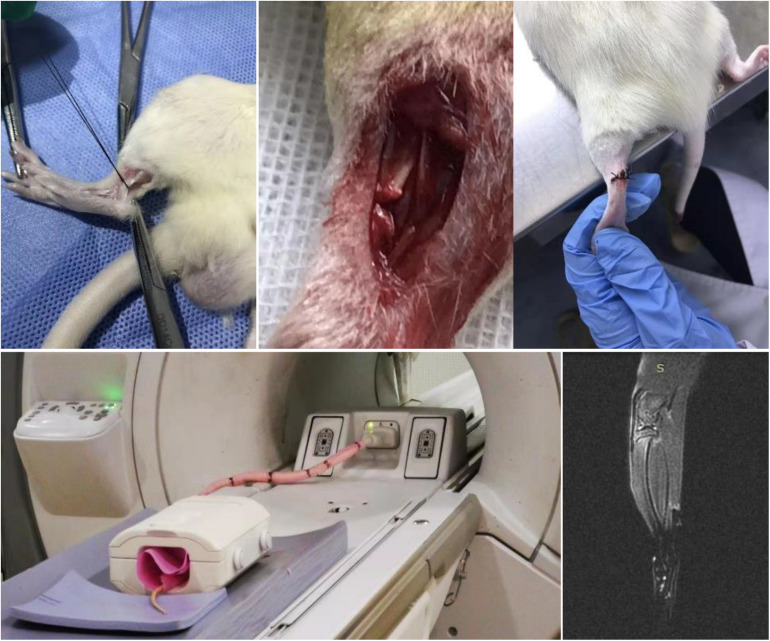
MRI image of rupture and repair of Achilles tendon. Under the aseptic conditions, Achilles tendon of rats were performed with line transverse incision and skin incision with the careful protection of blood vessels to prevent damage after vascular cavity, obtaining the Achilles tendon rupture model with 1–2 mm long transection of the defect. The successful modeling was determined by MRI image.

To better understand the basic cellular and molecular biology of tendon repair, gelatin/Fe_3_O_4_/celecoxib hydrogels were performed to assess the repair efficiency on the Achilles tendon of adult rats, and changes in macrophage phenotypes and related genes were analyzed. Compared with uninjured controls, injury tendon tissues were collected after 3, 7, 14, and 28 days, and the results found the accumulation of M1 macrophages in the early stage and then the transformation into the temporal and spatial distribution of M2 phenotype in Figure 5.

**FIGURE 6:**
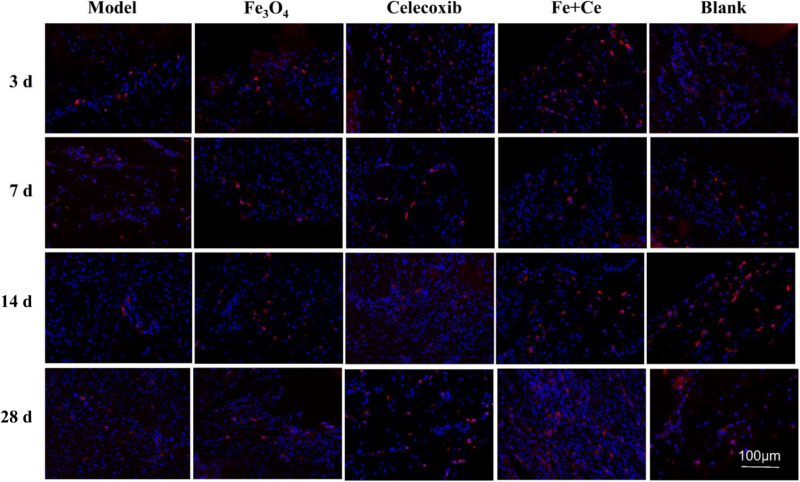
The spatiotemporal changes of M2 macrophages in the tendon tissues. Red dots represent M2 macrophages. After treatment for 3 days, 1, 2, 4 weeks, IF test showed the M2 macrophages with CD163 antibody markers (red) changed. All of the experimental groups appeared the red fluorescence from the 3-day and continue to 28 days, wherein the combined treatment group exhibited the highest proportion of M2 macrophages.

It is known that change in the phenotype of macrophages play an important role in the tendon repair, and M2 macrophages mainly exists in the reshaping of the late stage. After treatment for 3, 7, 14, and 28 days for the injured tendon tissue in rats, immunofluorescence technology test was investigated for evaluation of combination effects for tendon damage using M2 macrophages (CD163 antibody markers). The gelatin/Fe_3_O_4_/celecoxib sample showed that the number of M2 macrophages in all of groups were increased from the treatment of first 3 days, and achieve the highest proportion of M2 macrophages in the 2 weeks. In contrast, gelatin/Fe_3_O_4_ and gelatin/celecoxib groups exhibited similar increase trends, which were higher than the untreated control (Figure 6). The results suggested that the combined treatments could promote the increase in the proportion of M2 macrophages, thus accelerating the tendon repair process.

**FIGURE 7:**
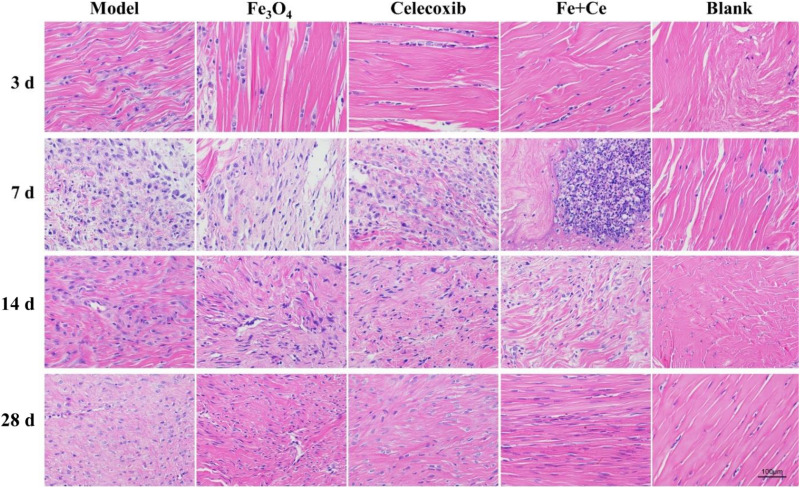
Pathological changes in tendon repair after combined treatment. H&E staining showed that inflammation from tendon rupture surgery began to invade with histopathology damage. After the treatment, all of the inflammation invasions were lower, wherein the combination treatment exhibited the lowest inflammatory invasion degree until the gradual recovery after 28 days, demonstrating the effective therapy to reduce the inflammatory response in process of tendon injury repair.

In addition, we also evaluated the combination therapy via the inflammatory infiltration and pathological injury of tendon injury site. Clinical studies found that inflammation existed in the process of tendon injury repair, and tendon healing was a complex and long process, including inflammation stage, proliferation stage and remodeling stage. H&E staining results showed that the control group exhibited serious tendon inflammation and histopathologic damage after the fracture operation invasion. After treatment for 3, 7, 14, and 28 days, gelatin/Fe_3_O_4_, gelatin/celecoxib and gelatin/Fe_3_O_4_/celecoxib groups could reduce the inflammation invasion, wherein the synergistic therapy showed the least inflammation invasion as shown in Figure 7. These results demonstrated that synergistic treatment can effectively reduce the inflammatory response in the process of tendon injuries repair.

### Gait Analysis Using Catwalk

**FIGURE 8:**
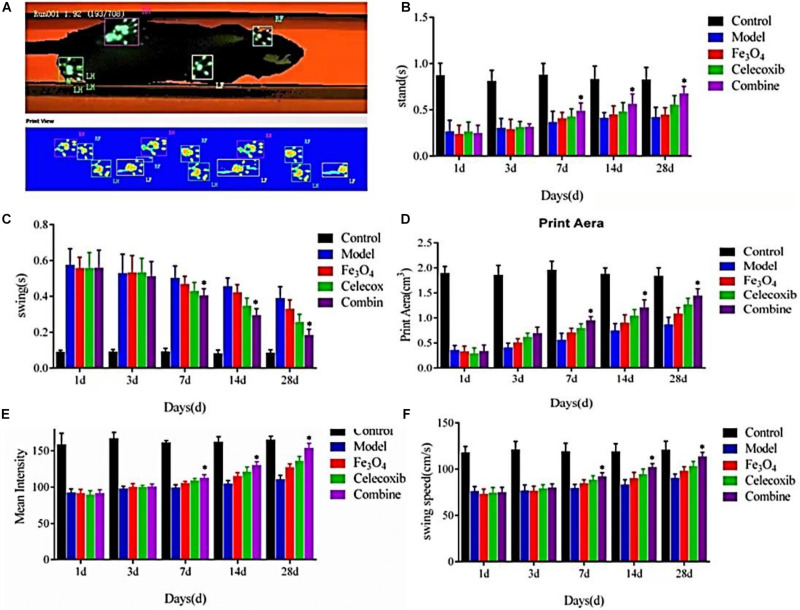
**(A)** CatWalk gait analysis of tendon injury repair of the rats. **(B)** Standing phase time, **(C)** swing phase time, **(D)** footprints area, **(E)** mean intensity, and **(F)** swing speed of the repaired rats (*P < 0.05).

The utility of gait analysis using CatWalk has been shown in various rodent models, such as sciatic nerve injury (Deumens et al., 2007), spinal cord injury (Hamers et al., 2006; Koopmans et al., 2005), myofascial inflammation (Miyagi et al., 2011), intervertebral disc injury (Miyagi et al., 2013), and inflammatory arthritis models (Ferreira-Gomes et al., 2008; Gabriel et al., 2007). It is well-known that injuries of limb joints and muscle tendons, tumors, malformations, neurological diseases and even psychological and mental states of the rats can affect the normal gait with various degrees. After the tendon injuries, the model rats can cause the long-term abnormal gait that may cause the secondary tendons damage and pains. Thus, the purpose of gait analysis in the present study was to identify the mechanism and causes of gait abnormalities, and treadmill running provide a platform and approach for gait analysis, which can obtain the quantitative and accurate gait data to provide the best treatment details and assess the rehabilitation efficacy. CatWalk gait analysis system, including the relevant parameters of standing phase time, swing phase time, footprints area, mean intensity and swing speed, can reflect the level of pain in rats and evaluate their tendon recovery, which provided important reference and indicators for the treatment and prognosis of tendon injuries after clinical trials. Therefore, we used the real-time Catwalk gait to analyze the tendon injury repair of the rats in Figure 8A.

As shown in Figures 8B–F, there were no significant differences in the standing phase time, swing phase time, footprints area, mean intensity and swing speed among all of the control group, model group, gelatin/Fe_3_O_4_ group, gelatin/celecoxib group and gelatin/Fe_3_O_4_/celecoxib group after the postoperative treatment for 1 and 3 days. After the operation for 7 days, the standing time, swing phase time, footprints area, mean intensity and swing speed of all of groups gradually increased. Until 28 days, the differences in the gelatin/Fe_3_O_4_/celecoxib group were statistically significant compared to other groups, indicating important roles in relieving the pain and promoting recovery prognosis. It was mentioned that gelatin/Fe_3_O_4_ and gelatin/celecoxib groups exhibited similar therapy effects, demonstrating the effects of pulsed electromagnetic field therapy on Achilles tendon rupture injury, which also further indicated the therapeutic combination of pulsed electromagnetic field and drug possessed great potentials in the rehabilitation of Achilles tendon rupture that can hopefully improve the walking function and promote tendon repair for the patients.

## Conclusion

In summary, we developed a new type of hydrogel dressing of gelatin/Fe_3_O_4_/celecoxib with combination therapies of celecoxib drug and pulse electromagnetic field for the injured tendon repair. The *in vitro* experiment revealed this gelatin/Fe_3_O_4_/celecoxib composite hydrogel system possessed functional characteristics and coordinated release behaviors under the external environmental stimuli. The *in vivo* evaluation showed a favorable repair by this synergistic strategy than that of single pulse electromagnetic or celecoxib drug, which not only effectively reduced the inflammatory reaction of macrophage cells but also contributed to the incremental proportion of M2 macrophages at the injury site. Furthermore, gait analysis revealed that synergistic therapy was important and effective to tendon injury repair in the later stage, that is, M2 macrophages played an active role in the production stage, and the polarization of M2-type macrophages was closely related to the repair of tendon injury. Therefore, this new type of combined tendon repair treatment will not only provide beneficially theoretical basis and therapeutic regimen for the clinical applications, but also reduce the economic burden and social pressure for the patients.

## Data Availability Statement

The data that supports the plots within this paper and other findings of this study are available from the corresponding authors upon reasonable requests.

## Ethics Statement

The animal study was reviewed and approved by Animal Ethics Committee of the Chinese PLA General Hospital. Written informed consent was obtained from the owners for the participation of their animals in this study.

## Author Contributions

LZ and YL initiated and designed the project, made suggestions, and revised the article. JW and LW collected the information and wrote this manuscript. YZ, YG, XH, TH, and BL checked and finalized the manuscript. All authors reviewed, commented, and approved the final manuscript.

## Conflict of Interest

The authors declare that the research was conducted in the absence of any commercial or financial relationships that could be construed as a potential conflict of interest.
